# A Review: The Bioactivities and Pharmacological Applications of *Polygonatum sibiricum* polysaccharides

**DOI:** 10.3390/molecules23051170

**Published:** 2018-05-14

**Authors:** Xiaowei Cui, Shiyuan Wang, Hui Cao, Hong Guo, Yujuan Li, Fangxue Xu, Mengmeng Zheng, Xiaozhi Xi, Chunchao Han

**Affiliations:** 1 School of Pharmacy, Shandong University of Traditional Chinese Medicine, Jinan 250355, China; 13553159162@163.com (X.C.); 13553158985@163.com (H.C.); m13589080261@163.com (H.G.); yjli_0531@163.com (Y.L.); xfx18364164925@163.com (F.X.); 18364166659@163.com (M.Z.); 18364167331@163.com (X.X.); 2 School of Traditional Chinese Medicine, Shandong University of Traditional Chinese Medicine, Jinan 250355, China; wsy0534@126.com

**Keywords:** *Polygonatum sibiricum* polysaccharides (PSP), compositions, pharmacological applications, biological activity, biosythesis, prospects

## Abstract

Traditional Chinese Medicine (TCM) has been widely used in China and is regarded as the most important therapeutic. *Polygonatum sibiricum* (PS), a natural plant used in traditional Chinese medicine, has various functions associated with a number of its components. There are many compositions in PS including polysaccharides, steroids, anthraquinone, alkaloids, cardiac glycosides, lignin, vitamins, various acids, and so on. Of these, polysaccharides play a significant role in PS-based therapeutics. This article summarizes *Polygonatum sibiricum* polysaccharides (PSP) have many pharmacological applications and biological activities, such as their antioxidant activity, anti-aging activity, an anti-fatigue effect, immunity enhancement effect, antibacterial effect, anti-inflammatory effect, hypolipidemic and antiatherosclerotic effects, anti-osteoporosis effect, liver protection, treatment of diabetes mellitus (DM), anti-cancer effect, and may help prevent Alzheimer’s disease, and so on. This review summarized the extraction method, purification method, compositions, pharmacological applications, biological activities, biosynthesis, and prospects of PSP, providing a basis for further study of PS and PSP.

## 1. Introduction

*Polygonatum sibiricum* (PS) is a dry rhizome of *Polygonatum kingianum* Coll. Et Hemsl., *Polygonatum sibiricum* Red., or *Polygonatum cytomema* Hua. which was introduced in the 2015 edition of pharmacopoeia. It is from the liliaceae plant which is distributed throughout the temperate Northern Hemisphere such as China, Japan, Korea, India, Russia, Europe, and North America [1,2,3]. Of these, China especially has abundant resources of PS. PS has a sweet fragrance and taste and is well-known as a traditional medicinal herb and functional food in China [4,5] as well as a health-improving substance [6]. PS has been reported to have many pharmacological applications and biological activities, such as antioxidant activity and anti-aging activity. It also has anti-osteoporosis, neuroprotective, immunity enhancement, anti-diabetic, anti-fatigue, and anti-cancer, effects [1,7,8,9]. PS has been used to treat fatigue, weakness, diabetes mellitus (DM), cough, indigestion, inappetence, sexual dysfunction, backache, and keen pain. Many publications have reviewed the chemical constituents, biological activities, and food use of PS [1,10]. The abundant biological activities have been attributed to the presence of multiple components, such as alkaloids, flavones, steroid saponins, lignins, amino acids, and polysaccharides (PSP) [5,8,11,12]. PSP are believed to be one of the most important active compounds of PS [13].

Water-soluble extractions of PSP, known for their low toxicity, are suitable for long-term administration for various activities and effects [1,5]. PSP were found to be the major contributors to the sweet taste of PS which makes the food more readily accepted [14]. At present, PSP are widely used in TCM therapy for cardiovascular diseases and other diseases [15,16,17,18,19,20]. In conclusions, PSP play an important role in PS, which is commonly used in people’s lives.

## 2. Chemical Constitutions of PSP

PSP, when extracted with hot water, were mainly composed of fructose, [21]. Some articles, through research of PSP chemical composition and weight determination, structure analysis, and functional activity testing, confirmed that PSP were composed of monosaccharides, which mainly include mannose (Man), galactose (Gal), glucose (Glc), fructose (Fru), rhammnose (Rha), arabinose (Ara), and galacturonic acid (GalA), as well as small amounts of xylose (Xyl) and glucuronic acid (GlcA). PSP were found that consisted of a branched homogalactan and branched galactomannans [1,3].

A study confirmed that crude PSP consisted of carbohydrates (85.1–88.3%) with proteins (4.51–11.9%) and uronic acid (1.79–7.47%) and included different percentage compositions of Man (62.3–76.3%), Glu (15.2–20.3%), Gal (4.35–15.3%), and Ara (4.00–7.65%) [4,5]. Another article indicated that Gal and Rha were the main components of PSP with little amounts of Man, Glu, and Xyl. It was confirmed that the ratio of Gal, Rha, Man, Glu, and Xyl were at 63.50%, 25.14%, 8.04%, 1.75%, and 15%, respectively [7].

## 3. Extraction and Purification Methods

The traditional extraction method of PSP is water extraction. PS was pretreated and extracted by using the hot water extraction method to get more PSP. Crude PSP were obtained, then ethanol precipitation was used to remove inactive substances and proteins [7]. The influencing factors in the process were extraction temperature, extraction frequency, extraction time, and solvent use. Chen Gang et al. [22] extracted PSP by a hot water extraction method optimized with the response surface method to get the optimal extraction condition. The result demonstrated that the optimal extraction conditions are as follows; solid to liquid ratio was 1:21.5, temperature was 73.5 °C, extraction time was 2.5 h. The extraction rate of PSP can reach 12.25% under these extraction conditions [23].

Dilute lye can help remove the chemical effects of plant cell wall molecules, then destroy the cell wall so that the active ingredient dissolves out of the cell, ultimately giving a higher yield of PSP [24]. The study demonstrated that the optimal conditions are as follows, crushing granularity was 60 mesh, alkali liquor was 3% NaOH solution, and the solid to liquid ratio was 1:15. The reaction it is then left overnight and then the final PSP extraction rate can reach 11.89%. However, the dilute alkali causes breakage of the glycosidic bonds of PSP. Therefore, the PH of the extracted liquid must be rapidly adjusted to avoid hydrolysis of PSP [23,24,25].

There are several other extraction methods for PSP, such as enzymatic hydrolysis extraction, ultrasonic crushing and extraction, microwave-assisted extraction, and so on [24]. Table 1 lists these methods.

## 4. Biological Activities

### 4.1. Antioxidant Activity and Anti-Aging Activity

PSP significantly reduces the malondialchehyche (MDA) content of skeletal muscle and serum, decreases free radical activity, and enhances the activity of superoxide dismutase (SOD) and glutathione peroxidase (GSH-Px) [19,25,26]. The article confirmed that PSP could protect human umbilical vein endothelial cells from damage induced by lipopolysaccharide [27]. At the same time, PSP can not only increase Na+-K+-ATP and Ca2+-ATP activity in mouse brain cells to prevent aging via Ca2+ overload, but it can also reduce the level of lipid peroxide (LPO), lipofuscin (LF), and B-type monoamine oxidase (MAO-B), thereby enhancing the body’s anti-damage and anti-aging effects [26,28]. Lu et al. demonstrated that there was a high correlation between aging and mitochondrial DNA damage and repaire genes during the aging process. PSP could confer an anti-aging effect through improving the energy metabolism of liver mitochondria, reducing the expression of DNA polymerase γmRNA and enhancing the activities of respiratory chain enzyme complexes [26]. Furthermore, PSP could enhance the antioxidant capacity of natural menopausal rats and improve their blood lipid metabolism to delay senescence [29]. Therefore, it plays an important role as an antioxidant and can delay senescence.

### 4.2. Anti-Fatigue Function

Recently, PSP has been studied as a new, natural anti-fatigue substance [30,31,32]. A previous study confirmed that PSP may have ergogenic and anti-fatigue functions [33]. Treatment with PSP decreases blood lactate and serum urea nitrogen and increases liver and muscle glycogen, all of which result in a reduction of fatigue [26,34]. Treatment with PSP could significantly prolong swimming time in mice. The results showed that PSP could improve the exercise endurance and enhance the fatigue resistance effect of mice [19,20].

### 4.3. Immunity Enhancement Effect

Some articles have shown that the immunologic functioning of mice could be increased by treatment with PSP [7,19,21,35,36,37,38]. For example, a PSP treatment group exhibited stimulation of natural red phagocytosis of RAW264.7 macrophages compared with the cyclophosphamide (Cy) treatment group. Furthermore, significantly accelerated recovery of spleen and thymus indices were recorded in mice [19]. PSP could enhance T and B cell proliferation responses as well as peritoneal macrophage phagocytosis. It could also restore the levels of interleukin-2 (IL-2), tumor necrosis factor (TNF-α), interleukin (IL-8), and IL-10 in serum which was treated with Cy in a dose-dependent manner. In conclusion, PSP could be used as an immunostimulant for protection against immunosuppression in Cy-treated mice [7].

The most recognized changes in the immune system were involution of the thymus and the diminished output of T lymphocytes [35]. The article found that the absolute number of total T cells (CD3+), involving both CD4+ and CD8+ subsets and the immune responses mediated by them, displayed an age-dependent decline [36]. Treatment with PSP significantly reversed the decline in the thymus and spleen weights, compared to the control group in Cy-induced immunosuppressed mice [1,37]. PSP can also promote the formation of hemolysin (an index of specific humoral immune function) and improve the phagocytic activity of peritoneal macrophages in immunosuppressed mice [1,38].

### 4.4. Antibacterial Effect and Anti-Inflammatory Effect

**Figure 1:**
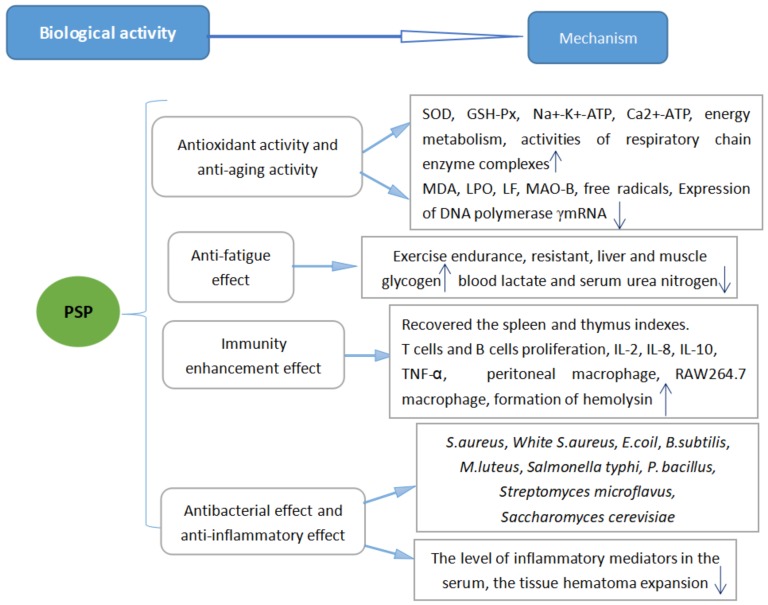
The biological activities of PSP and their mechanisms.

An experiment using the filter paper and cup method showed that PSP had antibacterial effects against *Staphylococccus aureus*, White *Staphylococcus aureus* (White *S. aureus*), *Escherichia coil*, *Bacillus subtilis*, *Salmonella typhi, Paratyphoid bacillus*, *Micrococcus luteus* [39], *Streptomyces microflavus*, and *Saccharomyces cerevisiae* [40]. Another experiment indicated that the minimal inhibition concentration (MIC) of PSP was 0.02 g/mL for *E. coil* and 0.01 g/mL for *S. aureus* and *M. luteus*. When PSP was extracted by the microwave-assisted method, the extraction rate of PSP could be as high as 10.57% [40]. Another experiment found that PSP could inhibit *E. coil*, *B. subtilis*, and *S. aureus*, with respective MICs of 1.23 mg/mL, 0.98 mg/mL, and 1.31 mg/mL [41]. From previous studies, PSP could also control ear swelling caused by xylene. The mechanism of these effects may involve reducing the level of inflammatory mediators in the serum, but the specific mechanisms still need further study [19]. Figure 1 shows all the known biological activity mechanisms of PSP.

## 5. Pharmacological Applications

### 5.1. Prevention of Alzheimer’s Disease (AD)

Neurodegeneration is a pathological condition of brain cells [42,43]. A study supposed that amyloid β-peptide (Aβ)-induced oxidative stress led to neurodegeneration in the AD brain [44]. An in vitro study showed that pretreatment with PSP could remarkably reduce the apoptotic rate and elevate the Bax/Bcl-2 ratio in rat PC12 cells and inhibit mitochondrial dysfunction and cytochrome release into the cytosol. Pretreatment with a P13K inhibitor could abolish the protective effects of PSP. In addition, Aβ25-35-induced Caspase-3 activation was inhibited and the protein levels of phosphorylated Akt (p-Akt) were enhanced in PC12 cells upon treatment with PSP. So, the protective effect of PSP against Aβ25-35-induced apoptosis in PC12 cells could be associated with the enhancement of P13K/Akt signaling [6]. Therefore, we unambiguously suggest that PSP can attenuate Aβ-induced neurotoxicity in PC12 cells. As we all know, Aβ-induced neurotoxicity is a major cause of AD [6]. Others have reported that PSP might have a potential therapeutic effect against AD [45]. According to these studies, PSP could be used to prevent and treat Alzheimer’s disease.

### 5.2. Treatment of Diabetes Mellitus (DM) 

According to former studies, Alloxan (ALX) and Streptozocin (STZ) could induce DM [26]. Another study investigated the mechanisms of how PSP treatment of DM [26]. Firstly, PSP decreased the blood glucose level and increased the thymus index, spleen index, and liver index in ALX-induced diabetic mice. At the same time, PSP could decrease the content of malondialdehyde (MDA), enhance the total superoxide dismutase (T-SOD) and glutathione peroxidase (GSH-Px) in blood serum, and enhance liver tissue of ALX-induced diabetic mice. Secondly, PSP could significantly affect the levels of fasting blood glucose (FGB), glycosylated serum protein (GSP), total cholesterol (TC), and triglyceride (TG) [36,46], and the amount of water drinking, food intake, and urinary volume in the PSP-treated groups were lower than the control group of STZ-induced diabetic mice. PSP decreased the rate of apoptotic cells, the levels of Caspase-3 and NO in blood serum, and iNOS mRNA expression in STZ-induced diabetic mice [26,47]. They found that the mechanism of the protective effect of PSP on ALX-induced diabetic mice might be related to its antioxidant activity. On the other hand, PSP decreased blood glucose and had some protective activity in STZ-induced diabetic rats. The mechanism of this may be related to inhibition of islet cell apoptosis, Caspase-3 reduction, and suppression of iNOS mRNA activity [26,48,49]. As PSP could decrease the TG and TC of hyperlipidemic animals, the mechanism may be related to its anti-oxidant effect, immune regulation, or inhibition of inflammatory factors [26].

One of the most common microvascular complications in DM is diabetic retinopathy (DR). A study confirmed that treatment with PSP not only could slow the progression of DR, but could also affect cataract formation through alleviating hyperglycemia and reducing oxidative stress in diabetic mice which induced by STZ [50]. Some previous studies found that compared to the normal rats, the structure of the gut microbiota was significantly changed in diabetic rats treated with PSP. Oral administration with PSP could prevent type II diabetes by its regulatory role on the gut microbiota [51]. Also, PSP-lowered blood sugar and lipids and alleviated fatty liver degeneration in type II diabetes may be associated with the lower expression of SREBP-1c and SCD-1 proteins [52]. PSP could improve myocardial dysfunction in diabetic rates which may be related to its promotion of the expression of bone morphogenetic protein-7 (BMP-7) in cardiac tissue that affected the transforming growth factor-β1 (TGF-β1)/Smads signaling pathway [53]. Another study demonstrated that PSP could produce protective effects on myocardial inflammation damage with type I diabetes through reducing blood sugar and lipids and inhabiting inflammatory reactions [54]. It could be used to protect the kidney of diabetic rats by reducing sugar and inhibiting fibrosis [55].

**Figure 2:**
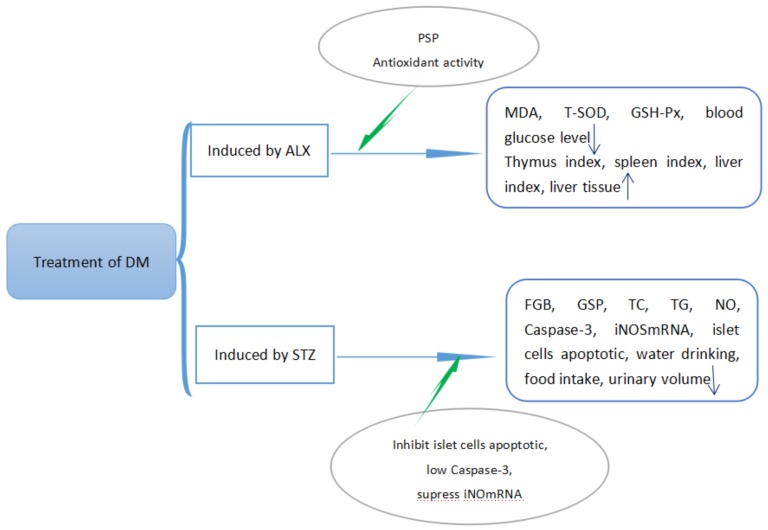
The mechanism of treatment of DM.

The process of PSP protection against DM is presented in Figure 2.

### 5.3. Hypolipidemic and Anti-Atherosclerotic Effects 

Some studies confirmed that the hypolipidemic activity of PSP was mainly due to modulation of TC, low-density lipoprotein (LDL-C), and lipoprotein (Lp (a)) in an atherosclerosis model. [16] PSP significantly reduced the intimal foam cells number compared to the control group. According to a study in endothelial cells (ECs), PSP not only affected EC proliferation, but also protected ECs from injury and apoptosis induced by H_2_O_2_ and lipopolysaccharide (LPS). In conclusion, the anti-atherosclerotic effect of PSP may be associated with its hypolipidemic activity, improving aortic morphology function, and reducing foam cells number [16].

Some articles showed that PSP had potential as a pharmaceutical or dietary adjuvant to treat hyperlipidemia and atherosclerosis [13,15]. They demonstrated that PSP caused significant improvements in serum lipid profile, apolipoproteins, and endothelial dysfunction parameters. The result showed that PSP had a protective effect against hyperlipidemia-induced atherosclerosis in hamsters [13].

### 5.4. Anti-Osteoporosis Effect

PSP has shown anti-osteoporosis effects and reverses bone loss in vivo [3,56]. The study confirmed that treatment with PSP could inhibit the receptor activation of nuclear factor-ΚB ligand (RANKL)-induced osteoclastogenesis and exert prophylatic protection against LPS-induced osteolysis in vivo. They found that PSP prevented osteoporosis through the Wnt/β-catenin pathway without affecting the bone morphogenetic protein (BMP) signaling pathway. PSP enhanced the osteogenic differentiation of bone marrow mesenchymal stem cells (BMSCs) by increasing the activity of alkaline phosphatase (ALP) and the expression of osteoblastic differentiation makers, such as runt-related transcription factor (RUNX2), bone gla protein (BGP) and the characteristic proteins of osteoblasts and the major sign of the start of mineralization (OCN) [5,57,58,59]. Another study indicated that PSP promoted osteoblastic differentiation and mineralization via the signal-regulated kinase (ERK)/glycogen synthase 3β (GSK-3β)/β-catenin signaling pathways [10]. Another experiment demonstrated that PSP could inhibit bone loss in ovariectomize rats and prevent osteoporosis by increasing BMP and basic fibroblast growth factor (bFGF) [60]. From all of these, we draw the conclusion that PSP could be used to treat osteoporosis.

### 5.5. Liver Protection

Jiang et al. showed that administration of PSP could increase rats’ final body weight, liver antioxidant enzyme activities (SOD, catalase (CAT), GSH-Px, and glutathione reductase (GR)), decrease serum aspartate aminotransferase (AST), alanine aminotransferase (ALT), alkaline phosphatase (ALP) activities, and liver MDA level which were induced by CCL_4_ treatment. It was recorded that PSP could decrease the toxicity of CCL_4_ in mice.

### 5.6. Anti-Cancer Effect

PSP could not only could significantly inhibit the growth of H22-transplantation tumor, stop the cells in the G0/G1 stage, and increase expression of caspases 3, 8, and 9, but could also inhibit human breast cancer cells (MCF-27) and Herps and Eac tumor masses [26,61]. An experiment found that PSP could hold human esophageal cancer ECA-109 cells, human gastric cancer HGC-27 cells, and human colorectal cancer HCT-8 cells in the S stage, promoting their apoptosis [3]. Some studies showed that PSP could inhibit S180 ascites tumor [23,26,62]. An article indicated that PSP could inhibit Hela cells, human breast cancer MDA-MB-435 cells, human leukemia HL-60 cells, and human lung cancer H14 cells [63]. According to former studies, the effect of PSP on cancer cells might be related to the inhibition of cancer-associated fibroblasts (CAFs). Han et al. found that PSP could inhibit the growth of prostate-CAFs without inhibiting the growth of normal fibroblasts. The important discovery was that PSP stimulated autophagy of prostate-CAFs and inhibited prostate-CAF growth, indicating a novel anti-cancer strategy involving the inhibition of the growth of prostate-CAFs [60].

Some articles indicated that the spleen index and thymus index increased in rats after treatment with PSP, so the anti-tumor effect may be associated with immunologic function [23,24,25,26,39,62,63].

**Figure 3:**
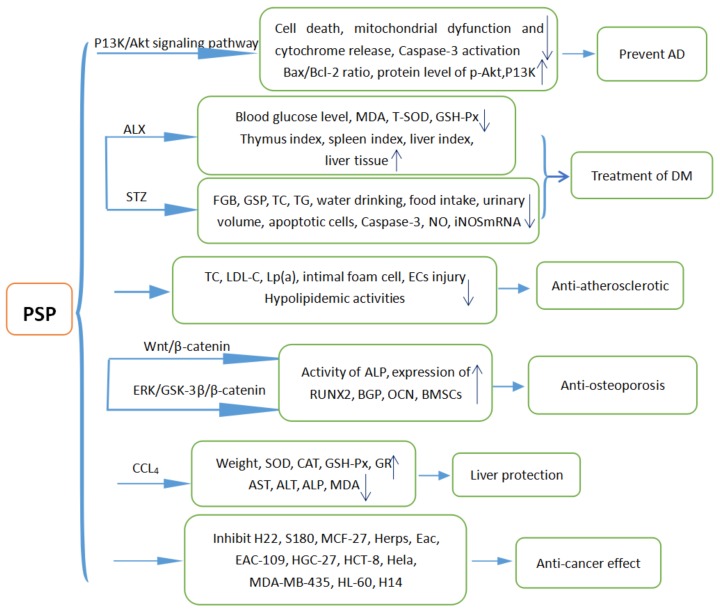
The pharmacological applications of PSP.

All of the pharmacological applications of PSP are shown in Figure 3.

## 6. Biosynthesis

**Figure 4:**
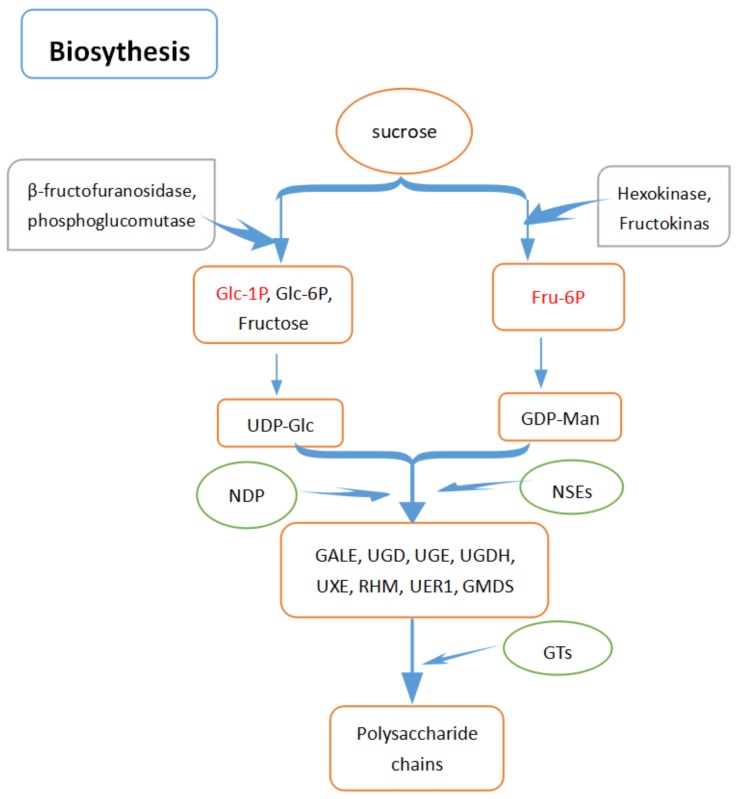
The biosynthesis of PSP.

Based on some studies [64,65,66,67,68,69,70,71,72], PSP biosynthesis can be divided into three main stages. Firstly, sucrose is converted to Glc-1P and Fru-6P. During these processes, many enzymes play important roles. β-fructofuranosidase (encoded by sacA) [66] converts sucrose to Glc-6P and Fructose, phosphoglucomutase (encoded by pgm) isomerizes Glc-6P to Glc-1P [67], and hexokinase (encoded by HK) [68] and fructokinase (encoded by scrK) [69] also take part in the biosynthesis of Fru-6P. Secondly, uridine diphosphate glucose (UDP-Glc) is derived from Glc-1P [70] and Fru-6P is indirectly converted to GDP-Man [71]. UDP-Glc is the key precursor. Based on UDP-Glc and guanosine diphosphomannose (GDP-Man), other nucleotide-diphospho-sugars (NDP-sugars) are further converted through the action of NDP-sugar interconversion enzymes (NSEs) [72], such as UDP-glucose 4-epimerase (GALE), UDP-D-galactose dehydrogenase (UGD), UDP-glucuronate 4-epimerase (UGE), UDP-glucose 6-dehydrogenase (UGDH), UDP-arabinose 4-epimerase (UXE), UDP-glucose 4,6-dehydratase (RHM), 3,5-epimerase-4-redutase (UER1), and GDP-mannose 4,6-dehydratase (GMDS). Finally, various NDP-sugars are obtained by growing polysaccharide chains by the action of glycosyltransferase (GTs) [14]. Figure 4 shows the biosynthesis process of PSP.

## 7. Future Prospects

With the growth of science and technology growth of the modern society, the pollution of the environment, and the intensification of competition, there is increasing pressure and worry. This results in an increasing level of sickness, particularly manifesting as immune decline, premature aging, cancer onset, and other sub-health phenomena. Thus, health and longevity become people’s goals, to maintain health and slow down the function of aging [26]. PSP, as a kind of medicine and food, is praised by the general consumer due to its various activities. In addition, China’s resources of PS are abundant, providing strong potential for its development and utilization. The study of PSP pharmacodynamics, chemical composition, and molecular structure are still incomplete. Therefore, more studies are needed in order to discover its chemical composition and the mechanisms of its biological functions in vivo and in vitro for its safe application in human health care [3].

Thus, more chemical constituents will be isolated from PS and their pharmaceutical activities will become better understood with the development of science and technology.

## 8. Conclusions

PS, as a TCM with broad prospects for development, has a long medical history in China. Study of PS is mainly concentrated on its steroid saponins and polysaccharides. PSP as the most important component of PS and has become more and more popular due to its various pharmacological applications and biological activities. It can be used to treat many clinical diseases, such as Alzheimer’s disease, hypolipidemia, atherosclerosis, osteoporosis, liver disease, diabetes mellitus (DM), and cancer. These pharmacological applications and bioactivities are associated with its structure. In terms of pharmacological activity, there is a lack of studies on monomeric compounds and their mechanisms of action. Therefore, we should pay attention to the extraction and site of activity to elucidate the basis of its pharmacophore, in order to provide the necessary scientific basis for its further development and utilization.

## Figures and Tables

**Table 1:** *Polygonatum sibiricum* polysaccharide (PSP) extraction methods.

Extraction Methods	Solid-liquid Ratio	Temperature (°C)	Time (h)	Extraction Rate (%)
Water extraction method	1:21.5	73.5	2.5	12.25
Dilute lye extraction method	1:15	Room temperature	12	11.89
Other extraction methods	Enzymatic hydrolysis extraction method, Ultrasonic crushing and extraction method, Microwave-assisted extraction			
